# Risk-Stratified Effectiveness of Cephamycins Versus Carbapenems for ESBL-Producing *Enterobacterales* Infections: A Systematic Review and Meta-Analysis

**DOI:** 10.3390/antibiotics15090935

**Published:** 2026-09-20

**Authors:** Giacomo Franceschi, Mattia Marchi, Samuele Cantergiani, Gabriella Orlando, Stefania Casolari, Erica Franceschini, Andrea Bedini, Cristina Mussini, Marianna Meschiari

**Affiliations:** 1 Department of Infectious Diseases, Azienda Ospedaliera-Universitaria of Modena, University of Modena and Reggio Emilia, 41121 Modena, Italy; 2 Hesperia Hospital, 41125 Modena, Italy; 3 Department of Biomedical, Metabolic and Neural Science, University of Modena and Reggio Emilia, 41121 Modena, Italy

**Keywords:** cephamycins, carbapenems, extended-spectrum β-lactamase, *Escherichia coli*, *Klebsiella pneumoniae*, bloodstream infection, urinary tract infection, meta-analysis, antimicrobial stewardship

## Abstract

**Background/Objectives:** Carbapenems remain the standard treatment for infections caused by extended-spectrum β-lactamase–producing *Enterobacterales* (ESBL-E), but their widespread use contributes to the emergence of carbapenem resistance. Cephamycins have been proposed as carbapenem-sparing alternatives because of their relative stability against ESBL-mediated hydrolysis; however, their clinical effectiveness remains uncertain. We aimed to compare the effectiveness of cephamycins versus carbapenems as definitive treatment for ESBL-E infections. **Methods:** We searched Scopus, PubMed, and Embase for studies comparing cephamycins with carbapenems for the treatment of ESBL-producing *Enterobacterales* infections, with the search conducted through January 2026. Observational studies and randomized trials comparing cephamycins (cefmetazole, flomoxef, cefoxitin) with carbapenems in adult patients with microbiologically confirmed ESBL-E infections were included. The primary outcome was all-cause mortality; secondary outcomes were clinical and microbiological cure. Risk of bias was assessed using ROBINS-I, and data were pooled using random-effects models. **Results:** Ten retrospective cohort studies (n = 653 for mortality) were included. Cephamycins were associated with a non-significant trend toward lower mortality compared with carbapenems (OR 0.48, 95% CI 0.22–1.03). Subgroup analyses suggested a potential benefit in *Escherichia coli* and urinary-source infections, whereas no advantage was observed in *Klebsiella pneumoniae* or non-urinary bloodstream infections. No significant differences were found for clinical or microbiological cure. The certainty of evidence was rated as very low. **Conclusions:** Cephamycins may represent a carbapenem-sparing option in carefully selected patients with low-risk ESBL-E infections, particularly urinary-source infections caused by *E. coli*. However, the current evidence is limited to retrospective observational studies with very low certainty and should be considered hypothesis-generating rather than practice-changing. Adequately powered randomized controlled trials are needed to confirm these findings.

## 1. Introduction

Extended-spectrum β-lactamase–producing *Enterobacterales* (ESBL-E), most commonly *Escherichia coli* and *Klebsiella pneumoniae*, represent a major global health threat. Their increasing prevalence is associated with limited therapeutic options, prolonged hospitalization, higher healthcare costs, and increased mortality [1,2]. Extended-spectrum β-lactamase (ESBL) enzymes—primarily belonging to the CTX-M, TEM, and SHV families—hydrolyze penicillins, third-generation cephalosporins, and monobactams, while generally sparing carbapenems and cephamycins [3].

Carbapenems are widely regarded as the most reliable agents for the treatment of severe ESBL-E infections because of their stability against ESBL-mediated hydrolysis and consistent clinical efficacy [4]. However, their widespread use has contributed to the global emergence of carbapenem-resistant organisms [5], highlighting the urgent need for effective carbapenem-sparing strategies [6].

Older β-lactam/β-lactamase inhibitors (BLICs), such as piperacillin–tazobactam, demonstrated inconsistent clinical performance, particularly in high-inoculum infections and bloodstream infections (BSIs), where discordance between in vitro susceptibility and clinical outcomes has been observed [7,8]. Novel agents, including ceftazidime-avibactam and ceftolozane-tazobactam, provide additional options but may be limited by cost, restricted indications, emerging resistance, and availability [9,10]. Therefore, identifying effective and safe alternatives among existing antibiotic classes remains a priority.

Cephamycins are a distinct subclass of β-lactam antibiotics structurally related to cephalosporins but characterized by the presence of a 7-α-methoxy group on the β-lactam ring [11]. This structural feature increases steric hindrance and enhances stability against hydrolysis by many β-lactamases, including ESBLs [12,13]. Agents in this class include cefoxitin (FOX), cefotetan, cefmetazole (CMZ), moxalactam, and flomoxef (FMOX).

Like other β-lactam antibiotics, cephamycins exert bactericidal activity through inhibition of penicillin-binding proteins, thereby disrupting bacterial cell wall synthesis. Cephamycins generally exhibit good activity against Enterobacterales, including *E. coli*, *K. pneumoniae*, *K. oxytoca*, and *Proteus mirabilis*, as well as against anaerobic bacteria, including *Bacteroides fragilis*, whereas *Pseudomonas aeruginosa* is typically resistant [11,14,15]. Owing to their relative stability against ESBL-mediated hydrolysis, many ESBL-producing *E. coli* and *K. pneumoniae* remain susceptible to cephamycins in vitro, supporting their potential role as carbapenem-sparing agents [16]. However, their activity may be reduced in the presence of additional resistance mechanisms, such as AmpC β-lactamase production [17], porin loss, and efflux pump overexpression [18].

Moreover, the efficacy of cephamycins may be compromised under conditions of high bacterial inoculum, raising concerns about their performance in severe infections characterized by a high bacterial burden, such as bloodstream infections (BSIs) or septic shock. Specifically, recent data showed that cephamycin activity against ESBL-producing *E. coli* and *K. pneumoniae* was reduced at high bacterial inocula, suggesting that the inoculum effect may not only compromise their efficacy but also potentially favor the survival and selection of resistant subpopulations [19].

Consequently, despite promising microbiological activity, the clinical effectiveness of cephamycins as alternatives to carbapenems remains uncertain.

To address this gap, we conducted a systematic review and meta-analysis comparing cephamycins with carbapenems for the treatment of ESBL-E infections. We aimed to evaluate their association with clinically relevant outcomes, including mortality, clinical cure, and microbiological cure, in order to assess whether cephamycins may represent a safe and effective carbapenem-sparing option in selected clinical scenarios.

## 2. Methods

### 2.1. Study Design and Reporting Standards

This systematic review and meta-analysis were conducted in accordance with the Preferred Reporting Items for Systematic Reviews and Meta-Analyses (PRISMA) 2020 guidelines [20]. The study protocol was prospectively registered in the International Prospective Register of Systematic Reviews (PROSPERO; registration number CRD420261305669).

### 2.2. Literature Search Strategy

Scopus, PubMed and EMBASE were searched on 31 January 2026 using the search string reported in Supplementary Table S1.

The search strategy was adapted for each database. No restrictions were applied regarding publication year. Only studies published in English were considered.

In addition, the reference lists of relevant reviews and included articles were manually screened to identify additional eligible studies.

### 2.3. Study Selection

All records identified through database searching were imported into a reference management software (Zotero, version 9.0.6), and duplicate records were removed prior to screening. Titles and abstracts were independently screened by two reviewers to assess eligibility. The full texts of potentially relevant articles were subsequently reviewed for inclusion by the same two reviewers working independently.

Deduplication was performed by checking trial identification number (where available) and date/place of enrollment to make sure each sample rather than publication was the unit of interest.

Disagreements at any stage of the selection process were resolved through discussion or consultation with a third reviewer.

### 2.4. Eligibility Criteria

Studies were included if they met the following criteria:

- Population: Adult patients with documented infections caused by ESBL-E.
- Intervention: Treatment with cephamycins (including FOX, CMZ, FMOX, cefotetan and moxalactam).
- Comparator: Treatment with carbapenems (including meropenem, imipenem-cilastatin, ertapenem, doripenem, or biapenem).
- Outcomes: Clinical outcomes such as mortality, clinical cure, treatment failure, relapse, or microbiological cure.
- Study design: Randomized controlled trials, prospective or retrospective cohort studies, or case–control studies.

Exclusion criteria included case reports or case series involving fewer than 10 patients, studies lacking a carbapenem comparator group, in vitro or animal studies, reviews, editorials, and conference abstracts, as well as studies with unclear outcome reporting and those in which patients received both the intervention and the comparator treatment.

### 2.5. Data Extraction

Data were independently extracted by two reviewers using a standardized data collection form.

Extracted variables included:

- Study characteristics (author, year, country, study design).
- Patient demographics and infection characteristics.
- Type and dosing regimen of cephamycin and carbapenem used. Data on dosing regimens, infusion duration, and renal dose adjustments were extracted when available.
- Clinical outcomes of interest.
- Duration of follow-up.

Discrepancies in data extraction were resolved by discussion within the research group.

When required data were not retrievable from the published reports, corresponding authors were contacted twice by email, with a two-week interval between attempts; if no response was received, the study was excluded due to insufficient information. 

### 2.6. Clinical Outcomes

The primary outcome of this meta-analysis was all-cause mortality, as reported in the individual studies. When multiple mortality time points were available (e.g., in-hospital, 14-day, or 30-day mortality), all mortality data were extracted. However, to avoid duplicating information from the same sample, in the main meta-analysis we considered only one mortality time point per study, selecting the time point closest to the median time frame across studies. Secondary outcomes included clinical cure and microbiological cure, as defined in the original studies. Clinical cure was generally defined as resolution or significant improvement of infection-related signs and symptoms at the end of therapy or at test-of-cure assessment, according to each study’s criteria. Microbiological cure was defined as documented eradication of the causative pathogen from follow-up cultures.

### 2.7. Risk of Bias Assessment

The risk of bias in randomized controlled trials was assessed using the Cochrane Risk of Bias tool.

Non-randomized studies were evaluated using the Risk of Bias in Non-randomized Studies—of Interventions, Version 2 (ROBINS-I V2) [21]. Risk of bias assessments were performed independently by two reviewers.

### 2.8. Certainty of Evidence Assessment

The certainty of the evidence for each outcome was evaluated using the GRADE (Grading of Recommendations Assessment, Development and Evaluation) [22]. The assessment was performed independently by two reviewers.

In GRADE, randomized controlled trials start at high certainty and observational studies start at low certainty. Certainty was then rated down based on the following domains: risk of bias, inconsistency, indirectness, imprecision, and other considerations (e.g., publication bias).

### 2.9. Statistical Analysis

Meta-analyses were based on random effects models [20]. For continuous outcome data, we calculated the Hedges’ g standardized mean differences (SMDs) and the corresponding 95% confidence intervals (95% CIs); for dichotomous outcome data, we calculated the pooled odds ratios (ORs) and the corresponding 95% CIs [21]. Given the presence of zero-event cells in some studies, dichotomous outcomes were synthesized using a binomial-logit generalized linear mixed model (GLMM) with a random study effect [23]. The results were summarized using forest plots. Standard Q tests and the I^2^ statistics (i.e., the percentage of variability in prevalence estimates attributable to heterogeneity rather than sampling error or chance, with values of I^2^ ≥ 75% indicating high heterogeneity) were used to assess between-study heterogeneity [24].

Subgroup comparisons were conducted on the primary outcomes according to: (1) origin of infection, i.e., Urinary Tract Infection (UTI) versus UTI-related Blood Stream Infection (BSI) versus non-UTI-related BSI; (2) bacterial species responsible for the infection.

Subgroups were defined a priori into three categories using a 75% cut-off: (1) predominantly non–UTI-r BSI (>75% of included infections) versus mixed BSI populations (25–75% non–UTI-r BSI); versus predominantly UTI-r BSI (>75% of infections originating from a urinary source); (2) predominantly *E. coli* (>75% of isolates identified as *E. coli*) versus mixed populations (25–75% *E. coli*, with the remaining isolates mainly *K. pneumoniae*) versus predominantly *K. pneumoniae* (>75% of isolates identified as *K. pneumoniae*).

Subgroup differences were assessed using random-effects models, and between-subgroup heterogeneity was evaluated through chi-square tests for subgroup differences.

To explore the potential impact of confounding due to non-randomized treatment allocation, baseline imbalances between treatment groups (cephamycin vs. carbapenems) were quantified at the study level for the characteristics provided in ≥3 studies, which included: (1) age; (2) sex; (3) intensive care unit admission; (4) diabetes; (5) chronic renal disease; (6) bladder catheter; (7) immunosuppression; (8) coronary-artery disease; (9) liver disease; (10) cerebrovascular disease; (11) mechanical ventilation; (12) cancer; (13) nosocomial infection. For each characteristic, a study-specific imbalance effect size was computed using meta-analyses (SMD for continuous variables; log (OR) for binary variables). If a statistically significant pooled imbalance was detected, each imbalance measure was entered separately as a moderator in a univariable meta-regression of the primary outcomes to assess whether baseline imbalance was a significant effect modifier.

Sensitivity analyses also included leave-one-out analyses to explore the influence of individual studies on pooled estimates.

For other continuous baseline variables (e.g., Body Mass Index, Pitt Bacteremia Score, Charlson Comorbidity Index), standardized mean differences could not be calculated because measures of dispersion were not consistently reported across studies. Therefore, pooled weighted means were computed descriptively for each treatment group.

Small-study effects and publication bias were explored visually using funnel plots and statistically using Egger’s regression test [25]. These analyses are generally considered informative when at least 10 studies contribute to a meta-analysis; otherwise, they should be considered exploratory [26]. If analyses showed a significant risk of publication bias, the trim-and-fill method was applied to estimate the number of missing studies and the adjusted effect size [27,28].

Analyses used the meta package [29] in R version 4.6.2 for MacOs[30]. Statistical tests were two-sided and used a significance threshold of *p*-value < 0.05.

### 2.10. Automation Tools

No automated screening tools were used for study selection. All records were screened manually by human reviewers. Therefore, no records were excluded by automation tools.

## 3. Results

### 3.1. Results of Study Selection

From January 2006 to January 2026, the literature search identified a total of 2310 records through database searching (Scopus n = 1347; PubMed n = 509; Embase n = 454). After removal of 486 duplicate records and 2 retracted publications, 1823 records were screened by title and abstract. Of these, 1792 were excluded for not meeting the inclusion criteria.

**Figure 1:**
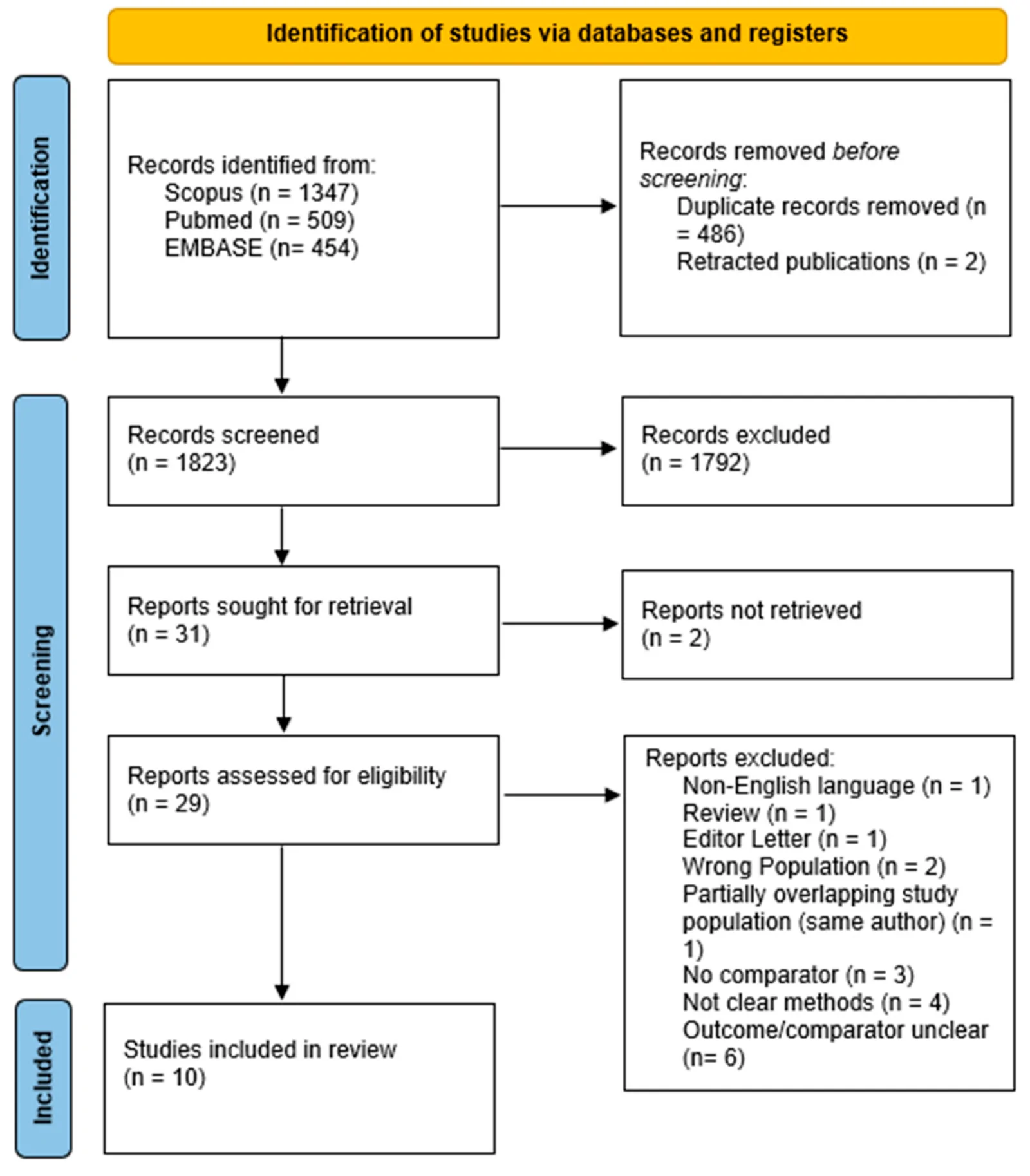
PRISMA 2020 flowchart representing the study selection process.

Thirty-one full-text reports were sought for retrieval; two could not be retrieved, and the remaining 29 reports were assessed for eligibility. Of these, 10 studies met the inclusion criteria and were included in the systematic review and meta-analysis. The detailed study selection process, including reasons for exclusion at each stage, is reported in the PRISMA 2020 flow diagram (Figure 1).

### 3.2. Study Characteristics

The main characteristics of the included studies are summarized in Table 1. All included studies were retrospective cohort studies published between 2006 and 2023; five of the ten included studies were conducted in Japan.

Overall, the studies included adult patients with infections caused by ESBL-E, predominantly *E. coli* (mean proportion of *E. coli* infections across the studies was 60.6%) and *K. pneumoniae* (mean proportion of *K. pneumoniae* infections across the studies was 32.0%). Infection types varied across studies and included uncomplicated and complicated urinary tract infections (UTIs), accounting for 27.2% of cases; UTI-related bloodstream infections (UTI-related BSIs), accounting for 33.4%; and bloodstream infections (BSIs) originating from non-urinary sources, accounting for the remaining 39.4% of cases. Several studies focused on specific clinical syndromes, such as febrile male UTIs, invasive UTIs, catheter-related BSIs, or intensive care unit (ICU) population.

The cephamycins evaluated included CMZ (55.5% of total cephamycin prescriptions), FMOX (26.5% of total cephamycin prescriptions), and FOX (18.0% of total cephamycin prescriptions), administered using heterogeneous dosing regimens, including high-dose strategies and, in some cases, continuous infusion. Carbapenem comparators consisted mainly of meropenem (56.5% of total carbapenem prescriptions), imipenem-cilastatin (22.4% of total carbapenem prescriptions) and ertapenem (19.6% of total carbapenem prescriptions).

Cephamycin dosing regimens varied across studies. Notably, the majority of studies did not specify the administered regimen (reported as “not reported”), and among those that did, FOX was generally administered at 2 g every 6–8 h (occasionally as continuous infusion), CMZ at 1 g every 6–8 h, and FMOX at 1 g every 6–8 h. Renal dose adjustments and infusion durations were inconsistently reported.

The severity of illness at infection onset varied substantially across studies. Some cohorts included predominantly stable patients with UTIs or low Pitt Bacteremia Score (PBS), whereas others enrolled critically ill patients with high rates of septic shock and universal ICU admission.

Definitions of mortality, clinical cure, and microbiological cure varied considerably across studies (Supplementary Table S2). Mortality outcomes included all-cause mortality, infection-attributable mortality within 14 days, and composite outcomes incorporating survival. Definitions of clinical cure exhibited substantial variation, encompassing (1) the resolution of infection-related signs and symptoms within 72 h or at the conclusion of therapy; (2) composite measures integrating symptom resolution with the absence of relapse and/or the necessity for antibiotic modification; (3) investigator-assessed clinical effectiveness; and (4) surrogate endpoints such as time to defervescence. Microbiological cure was generally defined as the documented eradication of the causative pathogen from follow-up cultures; however, the timing of follow-up cultures and the criteria for performing repeat cultures were not consistent across studies.

The weighted mean age was 72.8 years in the cephamycin group and 71.8 years in the carbapenem group. Mean BMI was comparable between groups (24.4 vs. 24.5, respectively). The weighted mean Charlson Comorbidity Index (CCI) was similar (3.5 vs. 3.6). In contrast, the weighted mean PBS was lower in the cephamycin group (2.4 vs. 3.5).

### 3.3. Primary Outcome: Mortality

**Figure 2:**
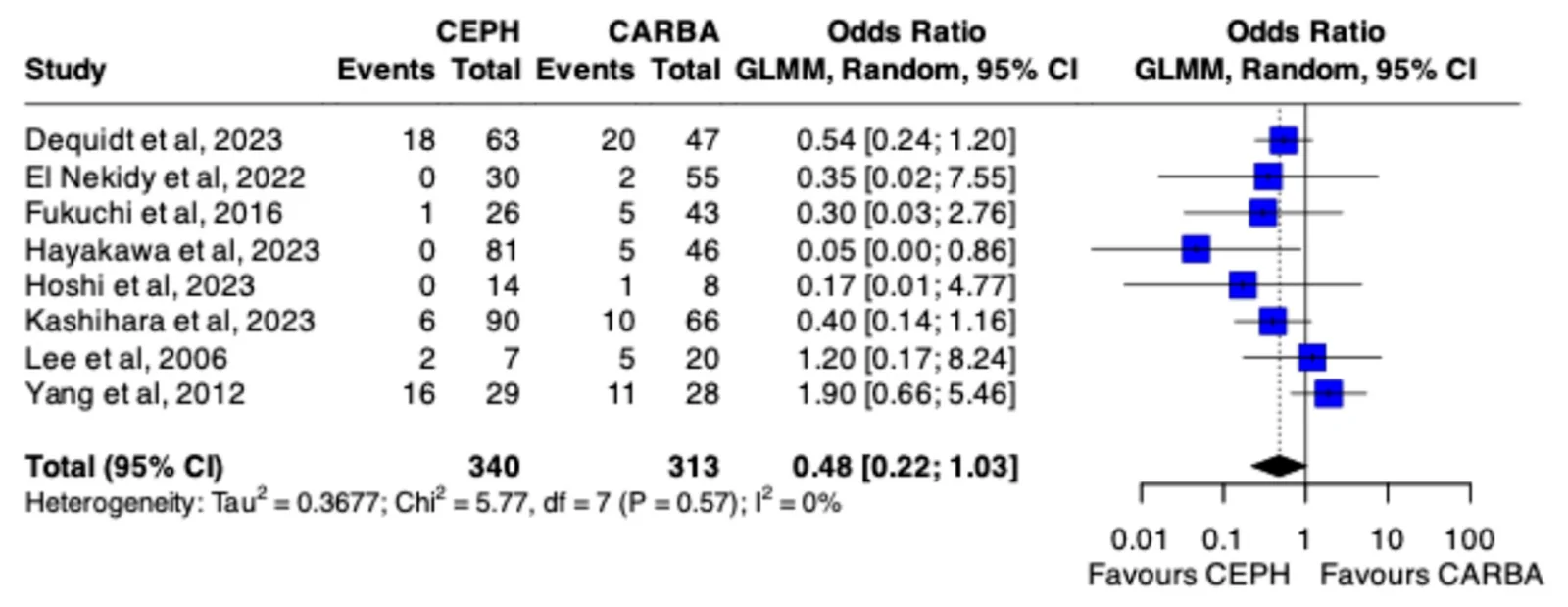
Forest plot of mortality (median follow-up time 30 days) among patients treated with cephamycin and carbapenem [references of the studies shown in the figure]. Blue squares represent individual study effect estimates, with square size proportional to study weight; black diamonds represent pooled effect estimates; the dashed vertical line represents the overall pooled effect estimate, and the solid vertical line at OR = 1 represents the line of no effect; **CARBA**: carbapenem; **CEPH**: cephamycin; **GLMM**: generalized linear mixed model; **95% CI**: 95% confidence interval.

Eight studies including 653 patients (340 treated with cephamycins and 313 with carbapenems) contributed to the mortality analysis. At the median follow-up time of 30 days, pooling data from two studies on 14 days mortality, one study on 90 days mortality, and the remaining five on 30 days mortality, cephamycins showed a non-statistically significant trend toward lower mortality compared with carbapenems (OR = 0.48 [95% CI: 0.22; 1.03] *p* = 0.059), with no evidence of between-study heterogeneity (I^2^ = 0%) (Figure 2).

**Figure 3:**
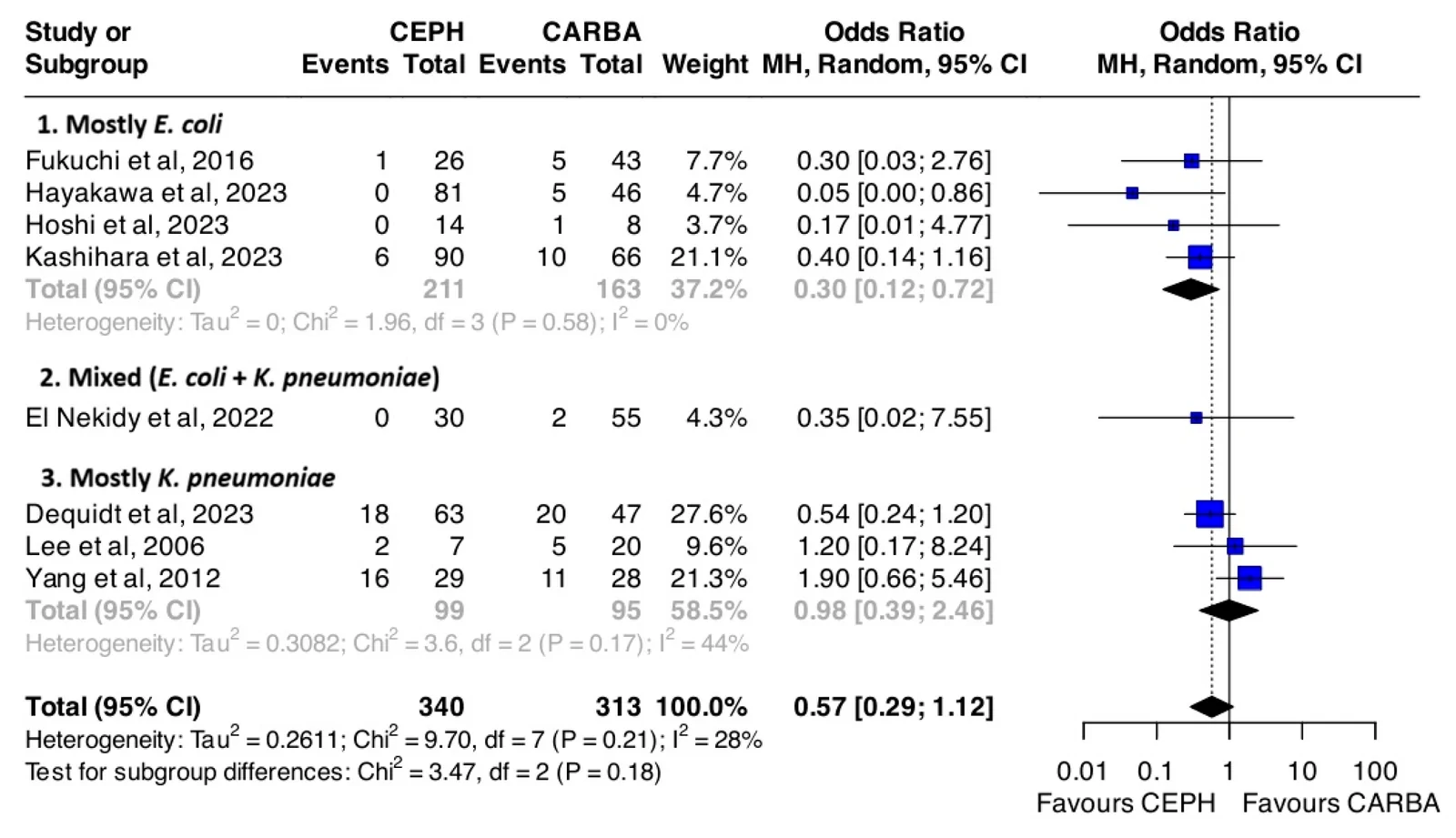
Forest plot of mortality (median follow-up time 30 days) among patients treated with cephamycin and carbapenem: overall and by bacterial species. Blue squares represent individual study effect estimates, with square size proportional to study weight; black diamonds represent pooled effect estimates; the dashed vertical line represents the overall pooled effect estimate, and the solid vertical line at OR = 1 represents the line of no effect. **CARBA**: carbapenem; **CEPH**: cephamycin; **EC**: Escherichia Coli; **KP**: Klebsiella pneumoniae; **MH**: Mantel-Haenszel; **95% CI**: 95% confidence interval.

**Figure 4:**
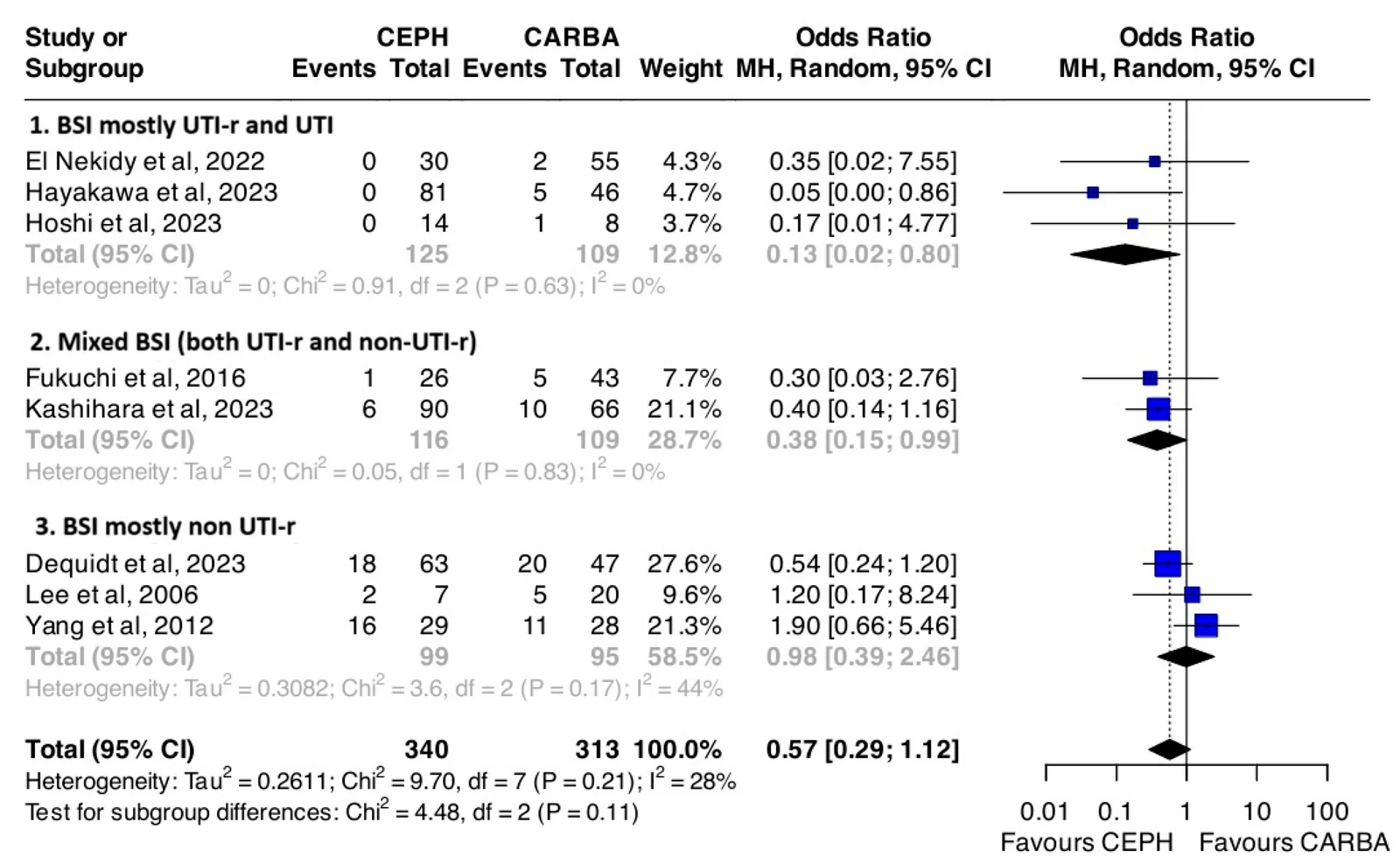
Forest plot of mortality (median follow-up time 30 days) among patients treated with cephamycin and carbapenem: overall and by infection type [references]. Blue squares represent individual study effect estimates, with square size proportional to study weight; black diamonds represent pooled effect estimates; the dashed vertical line represents the overall pooled effect estimate, and the solid vertical line at OR = 1 represents the line of no effect;**BSI**: blood stream infection; **UTI**: urinary tract infection; **BSI-UTIr**: blood stream infection related to urinary tract infection; **CARBA**: carbapenem; **CEPH**: cephamycin; **MH**: Mantel-Haenszel; **95% CI**: 95% confidence interval.

Subgroup analyses were conducted to assess potential effect modification according to bacterial species and infection source (Figure 3 and Figure 4).

In infections caused by *E. coli*, the direction of effect favored cephamycins (OR = 0.30 [95% CI: 0.12; 0.72]). However, when pooled analyses were expanded to include both *E. coli* and *K. pneumoniae*, and subsequently restricted to *K. pneumoniae* alone, the pooled estimates demonstrated a progressive shift in the pooled effect toward favoring carbapenems, although the difference remained statistically non-significant (OR = 0.98 [95% CI: 0.39; 2.46]). Despite the numerical differences in the pooled estimates, the test for subgroup differences was not statistically significant (*p* = 0.176)

### 3.4. Secondary Outcomes: Clinical and Microbiological Cure

A similar pattern emerged when analyses were grouped according to infection source. The mortality benefit observed in UTI-r BSI (OR = 0.13 [95% CI: 0.02; 0.80]) progressively diminished in mixed BSI populations (OR = 0.38 [95% CI: 0.15; 0.99]) and was further attenuated in cohorts predominantly composed of non–UTI-r BSI (OR = 0.98 [95% CI: 0.39; 2.46]). However, the test for subgroup differences was not statistically significant (*p* = 0.106).

**Figure 5:**
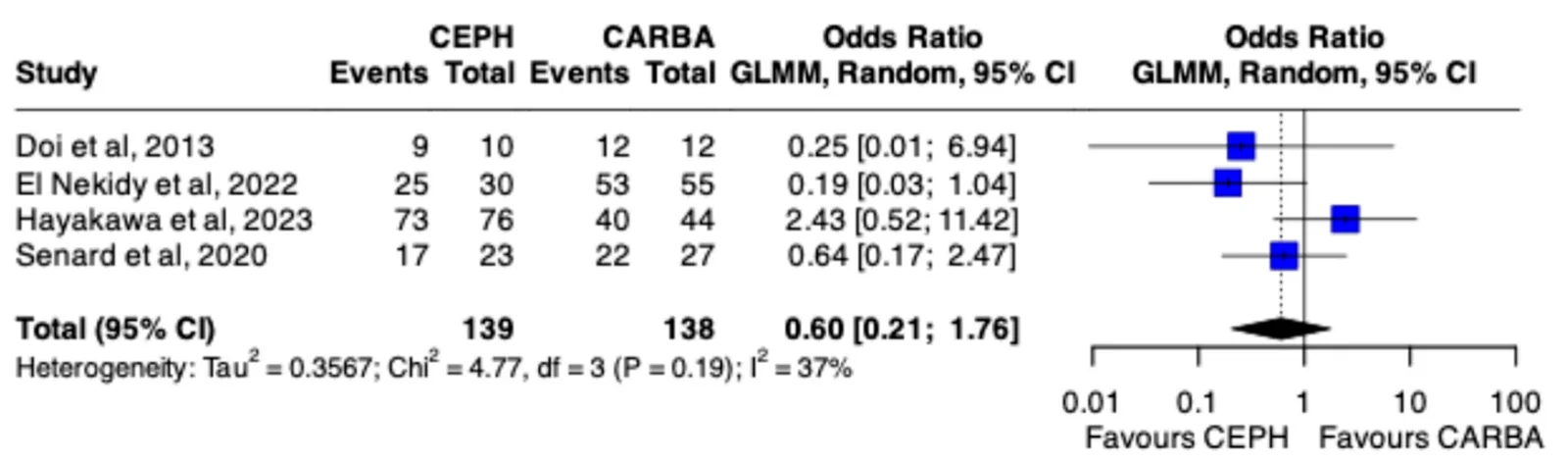
Forest plot of clinical cure (median follow-up time 30 days) among patients treated with cephamycin and carbapenem [references]. Blue squares represent individual study effect estimates, with square size proportional to study weight; black diamonds represent pooled effect estimates; the dashed vertical line represents the overall pooled effect estimate, and the solid vertical line at OR = 1 represents the line of no effect;**CARBA**: carbapenem; **CEPH**: cephamycin; **GLMM**: generalized linear mixed model; **95% CI**: 95% confidence interval.

**Figure 6:**
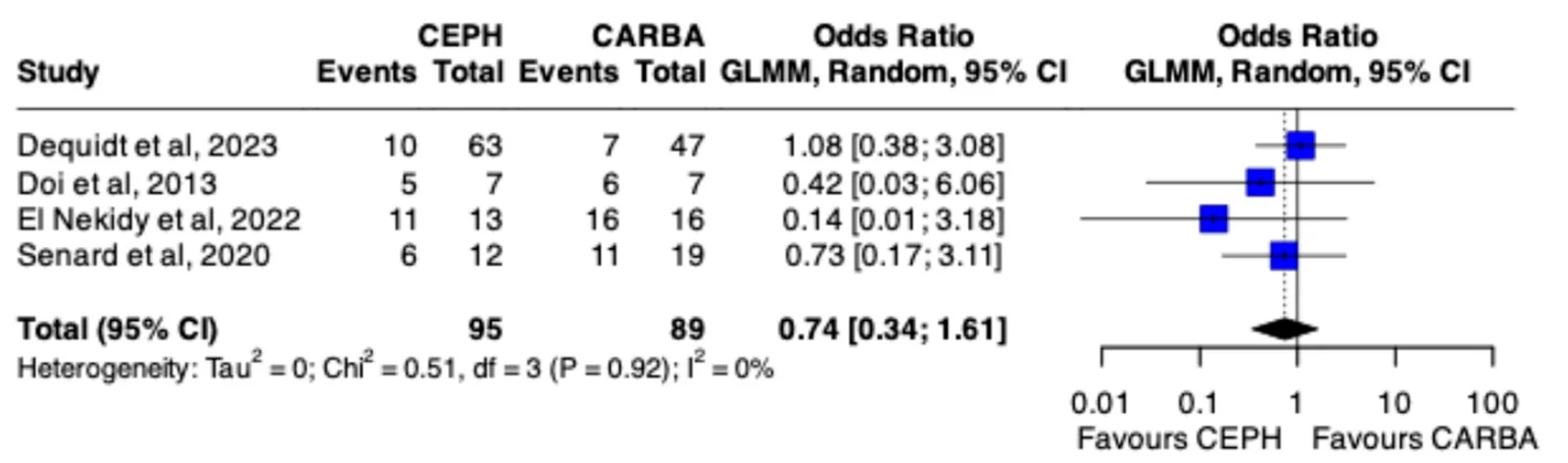
Forest plot of microbiological cure among patients treated with cephamycin and carbapenem [references]. Blue squares represent individual study effect estimates, with square size proportional to study weight; black diamonds represent pooled effect estimates; the dashed vertical line represents the overall pooled effect estimate, and the solid vertical line at OR = 1 represents the line of no effect; **CARBA**: carbapenem; **CEPH**: cephamycin; **GLMM**: generalized linear mixed model; **95% CI**: 95% confidence interval.

Figure 5 and Figure 6 illustrate the pooled analysis of secondary outcomes. Five studies (n patients = 299) evaluated clinical cure, yielding a pooled OR of 0.60 (95% CI: 0.21; 1.76, *p* = 0.352), with low-to-moderate heterogeneity (I^2^ = 37%).

Similarly, microbiological cure was assessed in five studies (n patients = 267), with a pooled OR of 0.74 (95% CI: 0.34; 1.61, *p* = 0.448) and no observed heterogeneity (I^2^ = 0%).

### 3.5. Confounder Meta-Analysis

To explore the potential impact of baseline imbalances due to non-randomized treatment allocation, we performed study-level meta-analyses of potential confounders (Supplementary Table S3). Significant differences between treatment groups were observed for ICU admission (OR = 0.34 [95% CI: 0.12; 0.96] *p* = 0.041), bladder catheter (OR = 0.52 [95% CI: 0.32; 0.84] *p* = 0.008), immunosuppression (OR= 0.41, [95% CI: 0.25; 0.69] *p* = 0.001), and cancer (OR = 1.93 [95% CI: 1.05; 3.53] *p* = 0.033). Specifically, patients treated with cephamycins were less frequently admitted to the ICU, less frequently immunosuppressed, and less likely to have bladder catheters, whereas cancer was more common in the cephamycin group. No statistically significant differences were observed for age, sex, diabetes, chronic renal disease, coronary artery disease, liver disease, cerebrovascular disease, mechanical ventilation, or nosocomial infection.

Univariable random-effects meta-regression analyses were performed to explore whether study-level baseline imbalances significantly modified the pooled mortality estimate. None of the tested moderators—ICU admission (β = 1.22 [95% CI: 0.365; 2.81] *p* = 0.131), immunosuppression (β = 3.32, [95% CI: 0.963; 7.61] *p* = 0.129), or cancer (β = −0.573, [95% CI: −5.55; 4.41] *p* = 0.822)—were significantly associated with the treatment effect (Supplementary Table S4). Bladder catheterization was not assessed in the meta-regression as it was reported only for two studies contributing to mortality meta-analysis.

### 3.6. Sensitivity Analysis (Leave-One-Out)

The leave-one-out analysis indicated that the statistical significance of the pooled mortality estimate was sensitive to the exclusion of individual studies, particularly Yang et al., whose exclusion resulted in a statistically significant association (OR 0.448, 95% CI 0.257–0.781), with negligible heterogeneity (I^2^ = 0%).

Sequential exclusion of other studies did not meaningfully change the direction or magnitude of the treatment effect for mortality, clinical cure, or microbiological cure (Supplementary Table S5).

### 3.7. Publication Bias

Publication bias was assessed using funnel plot inspection and Egger’s test. Given that only eight studies contributed to the mortality meta-analysis, these analyses were considered exploratory. Egger’s test was not statistically significant (*p* = 0.177); however, the small number of included studies limits the reliability and statistical power of this assessment, and publication bias cannot be reliably excluded. The funnel plot is reported in the Supplementary Figure S1.

### 3.8. Risk of Bias Results

The risk of bias assessment is summarized in Table 2.

According to the ROBINS-I V-2 tool, the included studies were judged to be at moderate to serious risk of bias. The overall assessment was primarily driven by risk of bias due to confounding, as treatment allocation in observational studies may be influenced by baseline prognostic factors and clinical severity. Studies lacking appropriate adjustment for baseline imbalances were rated at serious risk of bias, whereas those applying multivariable adjustment or propensity-based methods were rated at moderate risk. Bias related to participant selection and missing data was generally moderate, whereas bias in outcome measurement was largely at low risk. Overall, confounding represented the principal determinant of the ROBINS-I judgments across studies.

### 3.9. Certainty of Evidence Results

The certainty of the evidence for each outcome was evaluated using the GRADE framework. Overall, the certainty of evidence was rated as very low for all evaluated outcomes.

The main reasons for downgrading included the observational design of the included studies, the presence of moderate-to-serious risk of bias due to confounding, and imprecision of pooled estimates, as several confidence intervals included the line of no effect. In addition, the number of studies contributing to some pooled estimates was limited.

A detailed summary of the certainty assessment is reported in Supplementary Table S6.

## 4. Discussion

To our knowledge, this is the first meta-analysis specifically comparing cephamycins with carbapenems for the definitive treatment of infections caused by ESBL-E. Across ten retrospective observational studies, treatment with cephamycins was not associated with a statistically significant increase in mortality compared with carbapenems. Although the pooled point estimates numerically favored cephamycins, the confidence interval crossed the line of no effect, and the certainty of evidence was judged to be very low. Similar findings were observed for clinical and microbiological cure, with no statistically significant differences between treatment groups. Consequently, these findings should not be interpreted as evidence of equivalent efficacy or non-inferiority, but rather as hypothesis-generating observations that warrant prospective confirmation.

The growing interest in cephamycins reflects the urgent need to identify safe carbapenem-sparing strategies for the treatment of infections caused by ESBL-E, particularly as increasing carbapenem consumption may contribute to the emergence and dissemination of carbapenem-resistant organisms [5].

However, demonstrating the absence of a statistically significant difference in retrospective observational studies does not establish therapeutic equivalence. Treatment allocation in all included studies was based on clinical judgment rather than randomization, making residual confounding unavoidable despite the use of multivariable adjustment or propensity score analyses in several cohorts. Accordingly, our findings should be interpreted as describing the current body of observational evidence rather than providing definitive guidance for clinical decision-making.

An important consideration is the potential for confounding by indication. Patients receiving cephamycins were less frequently admitted to the ICU, less frequently immunosuppressed, and less likely to have bladder catheters, while the weighted mean Pitt Bacteremia Score was also lower in the cephamycin group (2.4 vs. 3.5). These differences suggest that clinicians may have preferentially selected cephamycins for clinically more stable patients, while reserving carbapenems for patients perceived to be at higher risk. Therefore, the apparent mortality advantage associated with cephamycins may be partly attributable to differences in baseline clinical severity rather than to the treatment effect itself. Although our study-level meta-regression did not identify statistically significant effect modification by the available confounders, this analysis cannot fully account for individual-level residual confounding.

Nevertheless, an important finding emerging from this meta-analysis is the consistent direction of the treatment effect across predefined subgroup analyses. The apparent benefit associated with cephamycins was progressively attenuated as the microbiological and clinical complexity of the study populations increased. This pattern may provide useful insights into the clinical settings in which cephamycins deserve further prospective evaluation.

In cohorts predominantly composed of ESBL-producing *E. coli*, cephamycins were associated with lower mortality, whereas this signal progressively disappeared in mixed populations and was no longer observed in cohorts enriched for *K. pneumoniae*. Although these subgroup findings should be interpreted cautiously because of the limited number of studies and patients, they are biologically plausible. *K. pneumoniae* differs substantially from *E. coli* in terms of virulence characteristics and resistance mechanisms, and several studies have demonstrated poorer clinical outcomes among patients with ESBL-producing *K. pneumoniae* BSIs compared with those caused by ESBL-producing *E. coli* [41,42]. Furthermore, *K. pneumoniae* more frequently harbors additional resistance determinants, including AmpC β-lactamases, porin alterations, and other mechanisms that may reduce cephamycin activity despite in vitro susceptibility [17,18,43]. Therefore, the attenuation of the treatment effect observed in *K. pneumoniae-*predominant cohorts is biologically consistent and further supports the need for pathogen-specific evaluation rather than considering ESBL-E as a homogeneous group.

A similar gradient was observed according to the source of infection. The most favorable treatment effect was consistently observed in UTIs and UTI-r BSIs, whereas no apparent advantage remained in cohorts dominated by BSI originating from non-urinary sources. Again, these observations should not be interpreted as evidence supporting preferential use of cephamycins in UTIs, but they suggest that the clinical context may substantially influence treatment performance. UTIs generally present lower bacterial inoculum, more favorable source control, and exceptionally high urinary antibiotic concentrations, all factors that may facilitate pharmacodynamic target attainment and improve treatment response [44]. Conversely, deep-seated infections and BSIs originating from non-urinary sources often involve a higher bacterial burden, impaired antibiotic penetration, and greater physiological instability, circumstances in which maximal antimicrobial activity becomes increasingly important.

Interestingly, these findings parallel the conceptual framework proposed by Gutiérrez-Gutiérrez and Rodríguez-Baño [45], who suggested that the appropriateness of carbapenem-sparing strategies should primarily depend on the overall clinical risk profile rather than solely on microbiological susceptibility. In their proposed approach, carbapenems remain the preferred therapy for patients presenting with severe sepsis, septic shock, or infections originating from high-risk sources, whereas alternative agents may be considered only in carefully selected patients with low-risk infections and favorable source control.

By “low-risk patients,” we refer to patients with clinical characteristics broadly corresponding to the populations in which cephamycins showed the most favorable signals in our analyses, particularly those with urinary-source infections caused by susceptible ESBL-producing E. coli and without features of severe systemic illness. This terminology is intended as a hypothesis-generating description rather than as a validated clinical risk classification. Although our meta-analysis was not designed to validate this strategy, the consistency between our subgroup analyses and this previously proposed risk-stratified framework provides additional biological plausibility to our observations.

Importantly, none of these subgroup findings should be regarded as sufficient to support changes in current clinical practice. All subgroup analyses were exploratory, involved relatively few studies, and were derived from non-randomized data. Rather than identifying patient populations in whom cephamycins should already replace carbapenems, our findings identify clinical scenarios that appear most suitable for future randomized controlled trials. Such trials should preferentially enroll carefully characterized low-risk patients, particularly those with urinary-source infections caused by susceptible ESBL-producing *E. coli*, in whom the potential benefits of carbapenem-sparing strategies may reasonably outweigh the risks associated with therapeutic uncertainty.

Following patient selection, optimization of antimicrobial exposure is likely to represent another key determinant of cephamycin effectiveness. Considerable heterogeneity was observed across the included studies regarding cephamycin dosing regimens, dosing intervals, duration of infusion, and adjustment for renal function, while pharmacokinetic and pharmacodynamic (PK/PD) data were inconsistently reported. Such variability deserves particular attention because cephamycins, similarly to other β-lactams, exhibit time-dependent bactericidal activity, making the proportion of the dosing interval during which free drug concentrations exceed the MIC (fT > MIC) the principal determinant of therapeutic efficacy [46,47].

Suboptimal drug exposure may be particularly problematic in critically ill patients, who frequently exhibit profound pharmacokinetic alterations, including augmented renal clearance, increased volume of distribution, and variable drug clearance. Likewise, isolates with MICs approaching the susceptibility breakpoint may require substantially higher exposures to achieve adequate pharmacodynamic targets. Previous PK/PD studies have demonstrated that conventional cefoxitin regimens may fail to achieve optimal target attainment under these circumstances, whereas higher daily doses or continuous infusion strategies may substantially improve the probability of pharmacodynamic success [48,49]. Nevertheless, exposure–response relationships could not be evaluated within the present meta-analysis because dosing strategies, therapeutic drug monitoring, and MIC distributions were inconsistently reported. Consequently, our findings should not be interpreted as supporting any specific dosing regimen. Future prospective studies should incorporate standardized PK/PD-driven dosing protocols and predefined pharmacodynamic targets to minimize treatment heterogeneity and allow a more robust evaluation of cephamycin efficacy.

Detailed PK/PD-informed dosing considerations for each cephamycin agent, stratified by renal function, are provided in Supplementary Table S7 [46,47,48,49,50,51,52,53].

From an antimicrobial stewardship perspective, identifying effective carbapenem-sparing strategies has become an increasingly important objective in the management of infections caused by ESBL-E. Carbapenems remain the treatment of choice for severe ESBL infections and constitute one of the most valuable antimicrobial classes currently available [4,54]. Their widespread use, however, contributes to selective pressure favoring the emergence and dissemination of carbapenem-resistant Gram-negative organisms. Consequently, stewardship programs increasingly aim not only to optimize patient outcomes but also to preserve the long-term effectiveness of last-resort antimicrobial agents.

Importantly, our findings should not be interpreted as supporting routine replacement of carbapenems by cephamycins. Rather, they suggest that cephamycins deserve further investigation as potential carbapenem-sparing agents in carefully selected low-risk clinical scenarios. This distinction is particularly relevant because the objective of antimicrobial stewardship is not indiscriminate de-escalation but the administration of the narrowest effective antimicrobial agent whenever this can be achieved without compromising patient safety [55,56].

This concept aligns with the recent perspective proposed by Murri and colleagues [57], who argued that antimicrobial stewardship should move beyond the traditional concept of “carbapenem sparing” and instead prioritize preservation of all agents classified as “Reserve” according to the WHO AWaRe classification [58]. Within this broader stewardship framework, cephamycins represent one of several potential therapeutic options that could reduce unnecessary exposure to broader-spectrum antibiotics. However, whether this theoretical advantage translates into improved clinical and ecological outcomes remains uncertain and requires prospective validation.

Beyond antimicrobial resistance, ecological considerations may further contribute to the rationale for evaluating cephamycins as alternative agents in selected patients. Carbapenems exert profound effects on the intestinal microbiota and have been associated with dysbiosis, colonization by multidrug-resistant organisms, and an increased risk of *Clostridioides difficile* infection [59]. Preliminary microbiome studies suggest that CMZ may induce less disruption of the intestinal microbial community than meropenem [60]. Although these observations are based on limited evidence and should be interpreted cautiously, they raise the possibility that narrower-spectrum agents such as cephamycins could offer ecological advantages extending beyond antimicrobial susceptibility alone. Future randomized trials should therefore incorporate ecological outcomes—including microbiome alterations, acquisition of multidrug-resistant organisms, and *C. difficile* infection—in addition to conventional clinical endpoints.

Finally, the potential role of cephamycins may extend beyond definitive treatment. Recent ESCMID/EUCIC guideline [61] recommends targeted perioperative prophylaxis for selected patients colonized with ESBL-producing *Enterobacterales* undergoing specific high-risk surgical procedures, while emphasizing that prophylactic regimens should be individualized according to local epidemiology and antimicrobial stewardship principles. Although current guidelines do not specifically recommend cephamycins for this indication, their microbiological activity against many ESBL-producing *Escherichia coli* isolates and their relatively narrow antibacterial spectrum make them biologically attractive candidates for future investigation. Whether cephamycins could contribute to reducing perioperative carbapenem exposure without compromising prophylactic efficacy remains unknown and represents another important area for prospective clinical research.

Overall, the available evidence should be viewed as an initial step toward defining a risk-stratified approach to carbapenem-sparing therapy rather than as evidence supporting a change in current treatment recommendations. Adequately powered randomized controlled trials remain essential to determine whether cephamycins can provide comparable clinical outcomes to carbapenems in carefully selected patients while simultaneously delivering meaningful antimicrobial stewardship and ecological benefits.

## 5. Limitations

Several limitations should be acknowledged.

All included studies were retrospective observational cohorts, making them inherently susceptible to residual confounding, particularly due to differences in illness severity influencing treatment choice and outcomes.

The limited number of studies in subgroup and meta-regression analyses reduced statistical power, rendering these analyses exploratory.

Although statistical heterogeneity was not detected for the mortality outcome (I^2^ = 0%), this finding should be interpreted cautiously given the limited number of included studies. Importantly, the absence of detected statistical heterogeneity does not imply clinical or methodological homogeneity. The included studies differed substantially in patient populations, infection sources, bacterial species, disease severity, cephamycin type and dosing strategies, mortality time points, and definitions of clinical and microbiological cure. This qualitative heterogeneity, particularly for clinical and microbiological cure outcomes, limits the confidence with which pooled estimates can be generalized across different clinical settings.

Moreover, outcome definitions were heterogeneous across studies, with mortality, clinical cure, and microbiological cure measured inconsistently, limiting comparability. Clinical heterogeneity was also substantial, including variation in infection sources, pathogen distribution, follow-up duration, and cephamycin dosing regimens, which may have affected pharmacodynamic target attainment and treatment efficacy.

Our analyses were based on published and registry summary data rather than individual participant data. As a result, subgroup analyses (particularly those between *E. coli*-predominant versus *K. pneumoniae*-predominant studies and urinary versus non-urinary infection cohorts) were performed at the study level and cannot establish that these variables actually modify the treatment effect at the patient level. As such, they provide only a relatively crude, hypothesis-generating analysis of differential effects.

Exposure–response relationships could not be formally assessed due to inconsistent reporting.

Finally, assessment of publication bias was necessarily exploratory because only eight studies contributed to the mortality analysis and the small number of studies and the predominance of Asian cohorts for CMZ and FMOX may limit the generalizability of the findings to other regions and resistance epidemiology contexts.

## 6. Conclusions

This systematic review and meta-analysis found no evidence of increased mortality associated with cephamycins compared with carbapenems for the definitive treatment of selected infections caused by ESBL-E. However, because all included studies were retrospective observational cohorts with moderate-to-serious risk of bias and the overall certainty of evidence was rated as very low, these findings should not be interpreted as evidence of comparable efficacy or non-inferiority.

The available evidence instead suggests that cephamycins may represent promising carbapenem-sparing agents for carefully selected low-risk patients, particularly those with UTIs or UTI-r BSIs caused by susceptible ESBL-producing *Escherichia coli*. Whether this strategy can safely reduce carbapenem exposure without compromising clinical outcomes remains uncertain.

Adequately powered randomized controlled trials incorporating standardized dosing strategies, microbiological characterization, PK/PD optimization, and ecological endpoints are now needed to determine the clinical role of cephamycins within a risk-stratified antimicrobial stewardship framework.

## Figures and Tables

**Table 1:** Basic characteristics of included studies.

Author (Year)	Country (Period of Study)	Population Characteristic	Cephamycin Group, n	Cephamycin Posology	Carbapenem Group, n	Carbapenem Posology	Source of Infection	Severity of Illness at Infection Onset	Organism(s)	Outcome(s)
Lee (2006) [31]	Taiwan (2004–2005)	BSI	7 (FMOX)	nr	20 (MEM = 14, IPM/CS = 6)	nr	Lung 55.6%; Intra-abdominal 18.5%; Urinary tract 11.1%; Soft tissue 3.7%; Other 11.1%	PITT score (mean): 5.3; ICU admission: 48.1%	*K. pneumoniae* (100%)	FMOX might be as clinically effective as a carbapenem in treating flomoxef-susceptible ESBL-BSI.
Yang (2012) [32]	Taiwan (2001–2007)	Nosocomial hemodialysis access-related BSI	29 (FMOX)	nr	28 (MEM = 12, IPM/CS = 16)	nr	HD catheter 73.7%; Fistula 12.3%; Graft 14.0	PITT score (mean): 5.3; ICU at the time of BSI: 47.6%	*K. pneumoniae* (100%)	Multivariate analyses revealed that FMOX use is associated with increased mortality
Doi (2013) [33]	Japan (2008–2010)	Pyelonephritis (50.0% pts w. urinary catheter inserted)	10 (CMZ)	nr	12 (MEM = 6, IPM/CS = 6)	nr	BSI UTI related 36.4%; Complicated UTI 63.6%	PITT score (mean): 1.92 (BSI only in carbapenem group); ICU data: nr	*E. coli* (91.3%) *K. pneumoniae* (4.3%) *P. mirabilis* (4.3%)	CMZ may be a useful option for the treatment of ESBL-UTIs.
Fukuchi (2016) [34]	Japan (2008–2013)	BSI	26 (CMZ)	nr	43 (MEM = 39, DOR = 3, BAPM = 1)	nr	BSI UTI related 50.7%; Source nr 49.3%	PITT score (mean): 2.12; ICU admission: 31.9%	*E. coli* (92.8%) *K. pneumoniae* (4.3%) *K. oxytoca* (2.9%)	CMZ may be a safe definitive option for ESBL-BSI, in stable patients.
Senard (2020) [35]	France (2013–2015)	Febrile male UTI (26.0% pts w. urinary catheter, 14.0% pts w. JJ stent)	23 (FOX)	6 or 8 g/24 h CIV	27 (IPM/CS = 13, ETP = 14)	IMP/CS: 1 g/8 h, ETP: 1 g/24 h	UTI 66.0%; severe sepsis 4.0%; septic shock 4.0%; BSI 26.0%	CCI (mean): 1.92 Severe sepsis: 4.0% Septic shock: 4.0%, ICU data: nr	*E. coli* (100%)	FOX is an effective treatment, particularly with high doses and CI
El Nekidy (2022) [36]	UAE (2015–2019)	UTI	30 (FOX)	2 g/6 h, ↓ 1–2 g/12–24 h in pts w/ impaired renal fxn	55 (ETP)	ETP: 1 g/24 h	UTI 100.0%	ICU at the time of therapy administration: 24.7%	*E. coli* (58.8%) *K. pneumoniae* (38.8%) Both (2.4%)	FOX achieved a microbiological cure rate similar to that of ETP
Hayakawa (2023) [37]	Japan (2020–2021)	Invasive UTI (39 pts w. urinary catheter inserted)	81 (CMZ)	CrCl > 50 mL/min: 2 pts 1 g/6 h; 1 pt 1 g/8 h; 11 pts 1 g/12 h; 4 pts 2 g/12 h; 2 pts 2 g/24 h.	46 (MEM)	CrCl > 50: 9 pts, 1 g/8 h, 2 pts: 0.5 g/12 h, 1 pt: 0.5 g/6 h, 1 pt: 0.5 g/8 h; 1 pt: 1 g/12 h	BSI UTI related 48.8%; UTI 51.2%	PITT score (mean): 3.0; ICU at the time of infection: 7.1%	*E. coli* (100%)	CMZ showed clinical and bacteriological effectiveness comparable to MEM
Dequidt (2023) [38]	France (Guadeloupe) (2013–2023)	BSI in ICU	63 (FMOX)	30 pts 6 g II; 17 pts 8 g II; 5 pts dose adj to renal fxn.	47 (MEM = 12, IPM/CS = 35)	nr	CRBSI 32.7%; Pneumonia 19.1%; Urinary 14.6%; Intra-abdominal 10.9%; Liver abscess 1.8%; Soft tissue 1.8%; Neuromeningeal 3.6%; Unknown 15.5%	All patient in ICU, w. BSI; PITT score (mean): 6.4; septic shock: 40.0%	*K. pneumoniae* (100%)	FOX as definitive therapy could be an option for some ESBL-BSI and reducing the selection of CR-*Pseudomonas aerugi nosa* strains.
Kashihara (2023) [39]	Japan (2014–2022)	BSI	90 (CMZ)	nr	66 (MEM = 62, IPM/CS = 3, DOR = 1)	nr	Urinary 65.4%; Intra-abdominal 11.5%; CRBSI 1.3%; Pneumonia 2.6%; Soft tissue 3.8%; Bone/arthritis 0.6%; Unknown 14.7%	PITT score (mean): 0.85; Immunocompromised patients: 26.3% ICU data: nr	*E. coli* (85.9%) *K. pneumoniae* (10.3%) *K. oxytoca* (0.6%) *P. mirabilis* (2.6%)	CMZ is a well-tolerated alternative to carbapenem for treating ESBL-BSI
Hoshi (2023) [40]	Japan (2018–2023)	BSI	14 (CMZ)	nr	8 (MEM)	nr	Urinary tract 77.3%; Pneumonia 9.1%; Other 13.6%	Patient with critical illness (included ICU admission) excluded; Pts w. cancer: 36.3%	*E. coli* (100%)	The delay in time to defervescence was greater with early CMZ administration than with MEM in patients with non-severe ESBL-BSI

**ESBL**: extended-spectrum β-lactamase; **BSI**: blood stream infection; **UTI**: urinary tract infection, **ICU**: intensive care unit; **FMOX**: flomoxef; **CMZ**: cefmetazole; **FOX**: cefoxitin; **MEM**: meropenem; **IPM/CS**: imipenem/cilastatin; **DOR**: doripenem; **BAMP**: biapenem; **ETP**: ertapenem; **II**: intermittent infusion; **CIV**: continuous infusion; **CrCl**: creatinine clearance; **pt(s)**: patient(s); **g**: gram, **HD**: hemodialysis, **CRBSI**: Catheter-related bloodstream infection, **CCI**: Charlson co-morbidity index; **CR**: carbapenem-resistance.

**Table 2:** ROBINS-I V2 assessment tool for included retrospective studies.

Author (Year)	D1	D2	D3	D4	D5	D6	Overall
Lee (2006) [31]	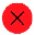	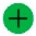	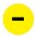	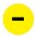	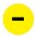	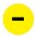	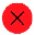
Yang (2012) [32]	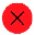	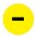	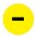	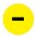	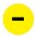	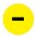	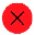
Doi (2013) [33]	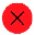	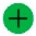	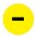	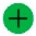	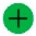	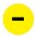	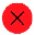
Fukuchi (2016) [34]	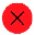	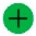	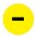	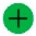	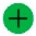	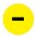	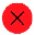
Senard (2020) [35]	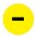	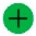	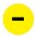	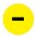	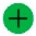	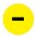	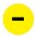
El Nekidy (2022) [36]	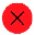	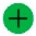	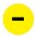	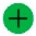	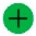	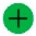	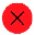
Hayakawa (2023) [37]	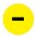	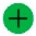	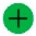	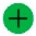	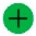	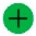	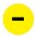
Dequidt (2023) [38]	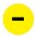	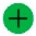	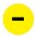	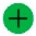	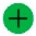	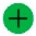	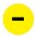
Kashihara (2023) [39]	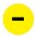	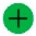	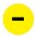	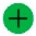	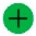	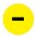	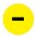
Hoshi (2024) [40]	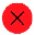	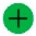	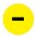	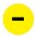	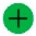	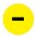	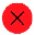

**Note:** Domain 1: Risk of bias due to confounding; Domain 2: Risk of bias in classification of interventions; Domain 3: Risk of bias in selection of participants into the study (or into the analysis); Domain 4: Risk of bias due to missing data; Domain 5: Risk of bias arising from measurement of the outcome; Domain 6: Risk of bias in selection of the reported result. Judgment: 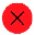 Serious; 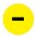 Moderate; 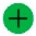 Low.

## Data Availability

All authors had full access to the data used in this study. Giacomo Franceschi is the guarantor of the data and takes responsibility for the integrity of the data and the accuracy of the data analysis.

## Supplementary Materials

The following supporting information can be downloaded at: https://www.mdpi.com/article/10.3390/antibiotics15090935/s1, Figure S1: Funnel plot of mortality meta-analysis; Table S1: Detailed Boolean search strings applied in Scopus, PubMed, and Embase for the identification of studies evaluating cephamycins compared with carbapenems for the treatment of infections caused by ESBL-producing Enterobacterales; Table S2: Definitions of mortality and clinical outcomes across included studies, highlighting variability in endpoint definitions, timepoints, and attribution criteria; Table S3: Meta-analyses of confounders among cephamycin and carbapenem groups; Table S4: Univariable meta-regression results of the mortality pooled estimate; Table S5: Leave-one-out meta-analysis of primary outcomes; Table S6: GRADE evidence summary for each outcome; Table S7: PK/PD-informed dosing regimens and exposure considerations for cephamycins in the context of future randomized controlled trials evaluating ESBL-E infections. References [46,47,48,49,50,51,52,53] are cited in Supplementary Table S7.

## Abbreviations

The following abbreviations are used in this manuscript:

ESBL	Extended-spectrum β-lactamase
ESBL-E	Extended-spectrum β-lactamase-producing Enterobacterales
BSI	Bloodstream infection
UTI	Urinary tract infection
ICU	Intensive care unit
CMZ	Cefmetazole
FOX	Cefoxitin
FMOX	Flomoxef
MEM	Meropenem
ETP	Ertapenem
IPM/CS	Imipenem/cilastatin
DOR	Doripenem
BAPM	Biapenem
OR	Odds ratio
CI	Confidence interval
CIV	Continuous intravenous infusion
GLMM	Generalized linear mixed model
ROBINS-I	Risk Of Bias In Non-randomized Studies of Interventions
GRADE	Grading of Recommendations Assessment, Development and Evaluation
PRISMA	Preferred Reporting Items for Systematic Reviews and Meta-Analyses
PK/PD	Pharmacokinetics/pharmacodynamics
MIC	Minimum inhibitory concentration
eGFR	Estimated glomerular filtration rate
CrCl	Creatinine clearance
TDM	Therapeutic drug monitoring
fT > MIC	Fraction of the dosing interval during which free drug concentrations exceed the MIC
T > MIC	Percentage of the dosing interval during which total drug concentrations exceed the MIC
CI (infusion)	Continuous infusion
EI	Extended/prolonged infusion
OPAT	Outpatient parenteral antimicrobial therapy
CRBSI	Catheter-related bloodstream infection
CCI	Charlson Comorbidity Index
