# Supplementary material for: Risk-Stratified Effectiveness of Cephamycins Versus Carbapenems for ESBL-Producing Enterobacterales Infections: A Systematic Review and Meta-Analysis
Source: Antibiotics (Basel). 2026 Sep 20;15(9):935. doi: 10.3390/antibiotics15090935 (PMC13603874; doi:10.3390/antibiotics15090935)
Supplement: Supplementary file 1 [file antibiotics-15-00935-s001.zip › antibiotics-4521778-Supplementary Table S2.pdf]

Supplementary Table S2. Definitions of mortality and clinical outcomes across included studies, highlighting variability in endpoint definitions, timepoints, and attribution criteria.

Study	Mortality Definition	Timepoint	Type of Mortality	Clinical Cure Definition	Microbiological Cure
Lee (2006)[31]	Death attributable to BSI	14 days from first BC	Infection-attributable	nr	nr
Yang (2012)[32]	Death attributable to BSI	14 days from first BC	Infection-attributable	nr	nr
Doi (2013)[33]	nr	-	-	Rate of clinical cure at 4 weeks after the completion of therapy	Rate of bacterial cure at the same point or thereafter if culture results were not available
Fukuchi (2016)[34]	Death within 30 days after onset of BSI	30 days	Infection-attributable	nr	nr
Senard (2020)[35]	nr	-	-	Resolution of signs and symptoms without recurrence both after 3 days from start of treatment and at follow-up visit.	Negative urine culture to the same ESBL-EC after 3 days from start of treatment and during follow-up.
El Nekidy (2022)[36]	Mortality within 90 days from index culture	90 days	All-cause (not explicitly specified)	Resolution of UTI symptoms within 72h and no need for additional antibiotics	Negative repeat culture during or immediately after end of therapy
Hayakawa (2023)[37]	30-day and in-hospital mortality	30 days / in-hospital	All-cause	Resolution or improvement of infection-related symptoms to baseline, assessed by ID specialist	Bacteriological response assessed but not uniformly defined
Dequidt (2023)[38]	30-day mortality	30 days	All-cause	nr	Microbiological failure defined as persistent ESBL-KP BSI ≥48 h and/or emergence of cefoxitin/carbapenem resistance during therapy or follow-up.
Kashihara (2023)[39]	90-day mortality	90 days	All-cause	nr	Absence of recurrent episodes of bacteremia
Hoshi (2023)[40]	30-day mortality	30 days	All-cause (not explicitly specified)	Time to defervescence used as surrogate clinical response	nr

BSI: bloodstream infection; **BC**: blood culture; **UTI**: urinary tract infection; **ID**: infectious diseases; **nr**: not reported; **ESBL**: extended-spectrum beta-lactamases; **EC**: *E. coli*; **KP**: *K. pneumoniae*.
