# Supplementary material for: Risk-Stratified Effectiveness of Cephamycins Versus Carbapenems for ESBL-Producing Enterobacterales Infections: A Systematic Review and Meta-Analysis
Source: Antibiotics (Basel). 2026 Sep 20;15(9):935. doi: 10.3390/antibiotics15090935 (PMC13603874; doi:10.3390/antibiotics15090935)
Supplement: Supplementary file 1 [file antibiotics-15-00935-s001.zip › antibiotics-4521778-Supplementary Table S5.pdf]

Leave-one-out analysis

Supplementary Table S5. Leave-one-out meta-analysis of primary outcomes

Author, year	OR (95%CI)	I^2	OR (95%CI)	I^2	OR (95%CI)	I^2
	Mortality		*Clinical cure*		*Microbiological cure*	
Dequidt et al, 2023[38]	0.541 (0.217; 1.35)	40%	NA	NA	0.591 (0.191; 1.83)	0%
Doi et al, 2013[33]	NA	NA	0.744 (0.219; 2.53)	44%	0.868 (0.389; 1.94)	0%
El Nekidy et al, 2022[36]	0.582 (0.287; 1.18)	36%	1.03 (0.372; 2.87)	8%	0.915 (0.414; 2.03)	0%
Fukuchi et al, 2016[34]	0.599 (0.291; 1.23)	36%	NA	NA	NA	NA
Hayakawa et al, 2023[37]	0.655 (0.346; 1.24)	25%	0.421 (0.159; 1.12)	0%	0.779 (0.355; 1.71)	0%
Hoshi et al, 2023[40]	0.600 (0.302; 1.19)	34%	0.622 (0.187; 2.07)	45%	NA	NA
Kashihara et al, 2023[39]	0.606 (0.266; 1.38)	35%	NA	NA	NA	NA
Lee et al, 2006[31]	0.512 (0.238; 1.10)	40%	NA	NA	NA	NA
Senard et al, 2020[35]	NA	NA	0.669 (0.138; 3.25)	46%	0.855 (0.344; 2.12)	0%
Yang et al, 2012[32]	0.448 (0.257; 0.781)	0%	NA	NA	NA	NA

Abbreviations (in alphabetical order): I^2: between-study heterogeneity; NA: not applicable; OR: odds ratio; 95%CI: 95% confidence interval.
