# Supplementary material for: Risk-Stratified Effectiveness of Cephamycins Versus Carbapenems for ESBL-Producing Enterobacterales Infections: A Systematic Review and Meta-Analysis
Source: Antibiotics (Basel). 2026 Sep 20;15(9):935. doi: 10.3390/antibiotics15090935 (PMC13603874; doi:10.3390/antibiotics15090935)
Supplement: Supplementary file 1 [file antibiotics-15-00935-s001.zip › Supplementary Table S1.pdf]

Supplement Table S1. Detailed Boolean search strings applied in Scopusm, PubMed and Embase for the identification of studies evaluating cephamycins compared with carbapenems for the treatment of infections caused by ESBL-producing *Enterobacterales*.

Scopus (n= 1347)	(((TITLE-ABS-KEY (esbl) OR TITLE-ABS-KEY (extended spectrum beta lactamase))) AND ((TITLE-ABS-KEY (cephamycins) OR TITLE-ABS-KEY (cephamycin) OR TITLE-ABS-KEY (cephamycin derivative) OR TITLE-ABS-KEY (flomoxef) OR TITLE-ABS-KEY (cefmetazole) OR TITLE-ABS-KEY (cefotetan) OR TITLE-ABS-KEY (cefoxitin))) AND ((TITLE-ABS-KEY (randomized controlled trial) OR TITLE-ABS-KEY (randomized controlled trial topic) OR TITLE-ABS-KEY (observational study) OR TITLE-ABS-KEY (controlled study)))) AND NOT (TITLE-ABS-KEY (animal))
Pubmed (n= 509)	(("beta-Lactamases"[Mesh]) OR ((extended spectrum beta lactamase) OR (esbl))) AND ((("Cephamycins"[Mesh]) OR ("flomoxef" [Supplementary Concept])) OR (flomoxef))
EMBASE (n= 454)	('extended spectrum beta lactamase'/exp OR 'esbl' OR 'extended spectrum beta lactamase') NOT ('animal'/exp OR 'animalia' OR 'metazoa' OR 'animal' OR 'animal population groups' OR 'animals' OR 'metazoan' OR 'metazoans' OR 'metazoon') AND ('cephamycin'/exp OR 'cefamycin' OR 'cephamycin' OR 'flomoxef'/exp OR '6315 s' OR '6315s' OR '7beta difluoromethylthioacetamido 7alpha methoxy 3 [[1 (2 hydroxyethyl) 1h tetrazol 5 yl] thiomethyl] 1 oxa 3 cephem 4 carboxylic acid' OR 'antibiotic 6315 s' OR 'antibiotic 6315s' OR 'cephalosporin 6315s' OR 'compound 6315 s' OR 'compound 6315s' OR 'flomoxef' OR 'flomoxef sodium' OR 'flumarin' OR 's 6315' OR 's6315' OR 'cephamycin derivative'/exp OR 'cefamycin derivative' OR 'cephamycin derivative' OR 'cephamycins' OR 'cefmetazole'/exp OR '7 [2 [(cyanomethyl) thio] acetamido] 7 methoxy 3 [[(1 methyl 1h tetrazol 5 yl) thio] methyl] 3 cephem 4 carboxylic acid' OR 'cefmetazole' OR 'cefmetazole sodium' OR 'cefmetazon' OR 'cefmetazone' OR 'cefometazole' OR 'cefotazole' OR 'ceftacin' OR 'cemetol' OR 'cetazone' OR 'cs 1170' OR 'cs1170' OR 'gomcefa' OR 'metalin' OR 'metazole' OR 'sodium cefmetazole' OR 'u 72791a' OR 'u72791a' OR 'zefazone' OR 'cefotetan'/exp OR '7 [4 (carbamoylcarboxymethylene) 1, 3 dithietane 2 carboxamide] 7 methoxy 3 [[(1 methyl 1h tetrazol 5 yl) thio] methyl] 2 cephem 2 carboxylic acid' OR 'apacef' OR 'apatef' OR 'cefotan' OR 'cefotan disodium' OR 'cefotetan' OR 'cefotetan and dextrose in duplex container' OR 'cefotetan disodium' OR 'ceftenon' OR 'cepan' OR 'ceph 2 em 2 carboxylic acid, 7 [4 (carbamoylcarboxymethylene) 1, 3 diethietane 2 carboxamido] 7 methoxy 3 [[(1

	methyl 1h tetrazol 5 yl) thio] methyl]' OR 'darvilen' OR 'ici 156, 834' OR 'ici 156834' OR 'yamatetam' OR 'yamatetan' OR 'ym 09330' OR 'cefoxitin'/exp OR '3 carbamoyloxymethyl 7 methoxy 7 (2 thenylacetamido) 2 cephem 2 carboxylic acid' OR '3 hydroxymethyl 7 methoxy 7 (2 thien 2 ylacetamido) 2 cephem 2 carboxylic acid carbamate ester' OR 'betacef' OR 'cefmore' OR 'cefotoxin' OR 'cefoxin' OR 'cefoxitin' OR 'cefoxitin and dextrose in duplex container' OR 'cefoxitin sodium' OR 'cefoxona' OR 'cefxitin' OR 'gamacef' OR 'l 620388' OR 'mefoxil' OR 'mefoxin' OR 'mefoxin in dextrose 5%' OR 'mefoxin in sodium chloride 0.9%' OR 'mefoxitin' OR 'merxin' OR 'mk 306' OR 'monowel' OR 'panafox' OR 'sodium cefoxitin' OR 'voxitin') AND ('randomized controlled trial (topic)'/exp OR 'pragmatic clinical trials as topic' OR 'randomized controlled trial (topic)' OR 'randomized controlled trials' OR 'randomized controlled trials as topic' OR 'controlled study'/exp OR 'control group study' OR 'control group trial' OR 'controlled study' OR 'controlled trial' OR 'observational study'/exp OR 'non experimental studies' OR 'non experimental study' OR 'nonexperimental studies' OR 'nonexperimental study' OR 'observation studies' OR 'observation study' OR 'observational studies' OR 'observational studies as topic' OR 'observational study' OR 'observational study as topic')
