# Supplementary material for: Risk-Stratified Effectiveness of Cephamycins Versus Carbapenems for ESBL-Producing Enterobacterales Infections: A Systematic Review and Meta-Analysis
Source: Antibiotics (Basel). 2026 Sep 20;15(9):935. doi: 10.3390/antibiotics15090935 (PMC13603874; doi:10.3390/antibiotics15090935)
Supplement: Supplementary file 1 [file antibiotics-15-00935-s001.zip › Supplementary Table S3.pdf]

**Supplementary Table S3.** Meta-analyses of confounders among cephamycin and carbapenem groups.

| Confounder | k | OR (95%CI)<br>SMD (95%CI) | p-value | $I^2$ |
|---|---|---|---|---|
| Age | 3 | -0.210 (-0.506; 0.086) | 0.164 | 13% |
| Number of male participants | 7 | 0.679 (0.401; 1.15) | 0.149 | 59% |
| ICU admission | 4 | 0.340 (0.120; 0.959) | **0.041** | 62% |
| Diabetes | 5 | 0.947 (0.326; 2.75) | 0.921 | 0% |
| Chronic renal disease | 4 | 0.810 (0.486; 1.35) | 0.419 | 0% |
| Bladder catheter | 4 | 0.516 (0.318; 0.838) | **0.008** | 0% |
| Immunosuppression | 5 | 0.414 (0.250; 0.686) | **0.001** | 0% |
| Coronary-artery disease | 3 | 1.23 (0.623; 2.42) | 0.552 | 0% |
| Liver disease | 3 | 1.24 (0.581; 2.64) | 0.578 | 19% |
| Cerebrovascular disease | 4 | 1.71 (0.985; 2.97) | 0.057 | 35% |
| Mechanical ventilation | 3 | 1.05 (0.228; 4.81) | 0.953 | 77% |
| Cancer | 4 | 1.93 (1.05; 3.53) | **0.033** | 0% |
| Nosocomial infection | 5 | 0.848 (0.509; 1.41) | 0.526 | 0% |

**CARBA**: carbapenem; **CEPH**: cephamycin; **$I^2$**: between-study heterogeneity; **ICU**: intensive care unit; **k**: number of studies contributing to the meta-analysis; **OR**: odds ratio; **SMD**: standardised mean difference; **95%CI**: 95% confidence interval.
Legend: For continuous variables, negative SMD (reported in *italics)* values indicate lower mean values in the cephamycin group compared with the carbapenem group. For binary variables, OR (regular font) values <1 indicate lower odds of the characteristic in the cephamycin group compared with the carbapenem group.
