# Supplementary material for: Risk-Stratified Effectiveness of Cephamycins Versus Carbapenems for ESBL-Producing Enterobacterales Infections: A Systematic Review and Meta-Analysis
Source: Antibiotics (Basel). 2026 Sep 20;15(9):935. doi: 10.3390/antibiotics15090935 (PMC13603874; doi:10.3390/antibiotics15090935)
Supplement: Supplementary file 1 [file antibiotics-15-00935-s001.zip › Supplementary Table S4 new.pdf]

Univariable meta-regression

Supplementary Table S4. univariable meta-regression results of mortality pooled estimate

Moderator	k	Beta (95%CI)	p-value
ICU admission	4	1.22 (-0.365; 2.81)	0.131
Immunosuppression	3	3.32 (-0.963; 7.61)	0.129
Cancer	3	-0.573 (-5.55; 4.41)	0.822

Abbreviations (in alphabetical order): ICU: intensive care unit; k: number of studies contributing to the analysis; 95%CI: 95% confidence interval.

Legend: univariable random-effects meta-regression of the (log) odds ratio for mortality (cephamycin vs carbapenem). Each moderator represents the study-level baseline imbalance between treatment groups-expressed as log(OR). Beta coefficients indicate the change in the log(OR) for mortality per 1-unit increase in the moderator; Beta values >0 indicate a shift toward higher mortality odds with cephamycin compared to carbapenems, and Beta values <0 indicate a shift toward lower mortality odds with cephamycin compared to carbapenems.
