# Supplementary material for: Risk-Stratified Effectiveness of Cephamycins Versus Carbapenems for ESBL-Producing Enterobacterales Infections: A Systematic Review and Meta-Analysis
Source: Antibiotics (Basel). 2026 Sep 20;15(9):935. doi: 10.3390/antibiotics15090935 (PMC13603874; doi:10.3390/antibiotics15090935)
Supplement: Supplementary file 1 [file antibiotics-15-00935-s001.zip › Supplementary Table S6.pdf]

Supplementary Table S6. GRADE evidence summary for each outcome

Certainty assessment							N of patients		Effect	Certainty	Importance
N of studies	Study design	Risk of bias	Inconsistency	Indirectness	Imprecision	Other considerations	Cephamycin	Carbapenem	OR (95%CI)		
Mortality											
8	Observational (retrospective cohort)	Serious	Not serious	Not serious	Serious	Not serious	340	313	0.48 (0.22; 1.03)	+??? VERY LOW	Primary
Clinical Cure											
4	Observational (retrospective cohort)	Serious	Not serious	Not serious	Serious	Serious	139	138	0.60 (0.21; 1.76)	+??? VERY LOW	Secondary
Microbiological cure											
4	Observational (retrospective cohort)	Serious	Not serious	Not serious	Serious	Serious	95	89	0.74 (0.34; 1.61)	+??? VERY LOW	Secondary

Abbreviations (in alphabetical order): N: number; OR: odds ratio; 95% CI: 95% confidence interval.

Explanation of reasons for downgrading/upgrading:

Evidence from observational studies started from low certainty.

We then GRADEd each pooled estimate for each relevant outcome according to the following criteria:

a. *Risk of Bias: We downgraded this domain by one level when any of the sources of Risk of Bias were rated as "high", or by half level when rated as "moderate", for any of the studies included in the pooled estimate.*

b. *Inconsistency: We downgraded this domain by one level where the I^2 value indicated substantial levels of heterogeneity (i.e., $I^2 \geq 75\%$).*

c. *Indirectness: We protected against indirectness by including studies directly evaluating the target population and intervention of interest. In addition, outcomes (mortality, clinical cure, and microbiological cure) were measured using direct clinical indicators rather than surrogate measures. Therefore, this domain was not downgraded.*

d. *Imprecision: We downgraded this domain by one level where the 95% confidence interval included the null value.*

e. *Other Considerations: We downgraded by one level when the number of studies contributing to the pooled estimate was <5.*
