# Supplementary material for: Risk-Stratified Effectiveness of Cephamycins Versus Carbapenems for ESBL-Producing Enterobacterales Infections: A Systematic Review and Meta-Analysis
Source: Antibiotics (Basel). 2026 Sep 20;15(9):935. doi: 10.3390/antibiotics15090935 (PMC13603874; doi:10.3390/antibiotics15090935)
Supplement: Supplementary file 1 [file antibiotics-15-00935-s001.zip › Supplementary Table S7 new.pdf]

Supplementary Table S7. PK/PD-informed dosing regimens and exposure considerations for cephamycins in the context of future randomized controlled trials evaluating ESBL-E infections. Dosing ranges are derived from published observational studies and PK/PD modeling analyses. These regimens were not formally compared within the present meta-analysis and are provided to support exposure standardization in future.

Cephamycin	eGFR ≥80 mL/min	eGFR 30–79 mL/min	eGFR <30 mL/min	MIC and PK/PD considerations
FOX	2 g q6h or 8 g/day CI	1–2 g q8h or 4–6 g/day CI	1 g q12–24h	CIV may be required to reliably achieve T>MIC 100% and T>4MIC 100% at higher MICs (≤8–32 mg/L) (1). 1-h infusion frequently inadequate at MIC ≥4 mg/L [48]. Suboptimal exposure associated with failure and resistance emergence in ICU setting [49]. Feasibility in OPAT reported [50].
CMZ	1 g q6h (2 g if MIC borderline)	1 g q8–12h	1 g q12–24h	Clinical data in ESBL-E invasive UTI show high efficacy with 1 g q8h over 1 h achieving median T>MIC ~92.6% for MIC ≤4 mg/L [51]. PK/PD simulations indicate optimal regimens vary with renal function to achieve ≥70% T>MIC targets [46]. Time-dependent activity underscores need for target attainment.
FMOX	1 g q6h (consider EI in 2h for higher targets)	1 g q8–12h	1 g q12–24h	Monte Carlo simulations suggest that bactericidal targets (e.g., ≥70% *f*T>MIC) are more consistently achieved with intensified regimens (e.g., 1 g q6h PI) in patients with higher eGFR [46]. Reduced penetration at some sites may warrant intensified exposure for deep infections. Site-specific PK in abdominal tissue indicates that q6h or q8h regimens can achieve ≥40% *f*T>MIC at MIC ~1 mg/L across tissues [47,52].PK/PD-guided optimization should be considered when MIC elevated [53].

eGFR: estimated glomerular filtration rate; CIV: continuous infusion; EI: prolonged infusion MIC: minimum inhibitory concentration; PK/PD: pharmacokinetic/pharmacodynamic; *f*T>MIC: free drug concentrations above the minimum inhibitory concentration; T>MIC: percentage of the dosing interval during which total drug concentrations exceed the minimum inhibitory concentration; ICU, intensive care unit; OPAT: outpatient parenteral antimicrobial therapy; q6h,8h,12h,24h: every 6, 8, 12, 24 hours.
